# Statistics

**Published:** 1895-06

**Authors:** 


Statistics of all countries show that unmarried men are far more liable to insanity than benedicts.

